# LLMDiff: Diffusion Model Using Frozen LLM Transformers for Precipitation Nowcasting

**DOI:** 10.3390/s24186049

**Published:** 2024-09-19

**Authors:** Lei She, Chenghong Zhang, Xin Man, Jie Shao

**Affiliations:** 1 Sichuan Artificial Intelligence Research Institute, Yibin 644000, China; leishe519@163.com (L.S.); manxin@std.uestc.edu.cn (X.M.); 2 Institute of Plateau Meteorology, China Meteorological Administration, Chengdu 610299, China; ipmzhang@gmail.com; 3 Intelligent Terminal Key Laboratory of Sichuan Province, Yibin 644000, China; 4 Shenzhen Institute for Advanced Study, University of Electronic Science and Technology of China, Shenzhen 518110, China

**Keywords:** precipitation nowcasting, image sequence prediction, diffusion model, radar echo map, large language model

## Abstract

Precipitation nowcasting, which involves the short-term, high-resolution prediction of rainfall, plays a crucial role in various real-world applications. In recent years, researchers have increasingly utilized deep learning-based methods in precipitation nowcasting. The exponential growth of spatiotemporal observation data has heightened interest in recent advancements such as denoising diffusion models, which offer appealing prospects due to their inherent probabilistic nature that aligns well with the complexities of weather forecasting. Successful application of diffusion models in rainfall prediction tasks requires relevant conditions and effective utilization to direct the forecasting process of the diffusion model. In this paper, we propose a probabilistic spatiotemporal model for precipitation nowcasting, named LLMDiff. The architecture of LLMDiff includes two networks: a conditional encoder–decoder network and a denoising network. The conditional network provides conditional information to guide the denoising network for high-quality predictions related to real-world earth systems. Additionally, we utilize a frozen transformer block from pre-trained large language models (LLMs) in the denoising network as a universal visual encoder layer, which enables the accurate estimation of motion trend by considering long-term temporal context information and capturing temporal dependencies within the frame sequence. Our experimental results demonstrate that LLMDiff outperforms state-of-the-art models on the SEVIR dataset.

## 1. Introduction

Precipitation nowcasting aims to predict rainfall in a localized area within 0∼2 h [1]. Predicting rainfall plays a critical role in preventing natural disasters such as mudslides and floods. Moreover, it may assist relevant personnel in efficiently managing water resources and fostering the growth of crops. Precipitation nowcasting can be regarded as the prediction of spatiotemporal sequence, utilizing radar echo maps with enhanced spatial resolution as the input [2,3]. Rainfall intensity and a forecast radar map are mutually converted through the Z-R relationship for precipitation nowcasting. Despite the utilization of data-driven algorithms, accurately predicting future rainfall remains a challenge due to the intricate nature of the atmosphere and nonlinear cloud dynamics.

The mainstream of previous research primarily centered around deterministic predictive models [1,2,3,4,5,6,7]. However, these models usually generate blurry predictions and estimate an inaccurate motion trend due to the Earth’s climatic system exhibiting high variability and complexity. Furthermore, the subtle differences in the initial state based on deterministic predictive models can lead to widely divergent performances that pose challenges to accurate prediction. The existing spatiotemporal deterministic models often cannot analyze or model the inherent nature of these differences. Consequently, their performance proves inadequate for precipitation nowcasting tasks. Deterministic models utilize convolutional recurrent neural networks, transformers, or their variants to predict the future sequence of radar echoes, involving direct optimization of the distance or similarity to the ground truth. While these models effectively capture dynamic trends, the forecast becomes increasingly blurry over time. Moreover, a common tendency arises in estimating the high-value echoes related to severe storms.

**Figure 1:**
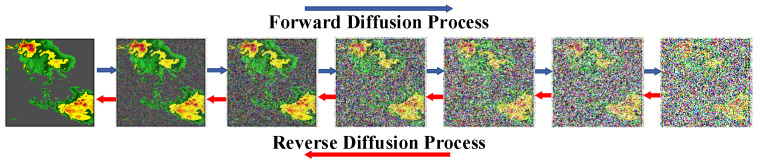
The diffusion process includes both forward (left to right) and reverse directions.

To address the issue of the inherent uncertainty in rainfall systems, researchers are currently exploring diffusion models (DMs) based on probabilistic predictive models. These models aim to provide more accurate and detailed forecasts by incorporating probabilistic techniques into the prediction process. As depicted in Figure 1, the forward process of the diffusion model adds noise to an image, while the reverse process removes noise to generate the original image. DMs have demonstrated impressive abilities in producing high-quality images [8,9] and videos [10,11]. Compared with generative adversarial networks (GANs) [12,13], being a likelihood-based model, DMs avoid mode collapse or training instabilities. The DMs’ approaches can generate more rapidly and with superior quality. They excel in enhancing their precision by generating realistic future details. Following Lu et al. [14], only by acquiring worldly knowledge, including spatiotemporal relationships and physical principles, can the model generate the future corresponding to the real world.

Despite the successful utilization of DMs in the generation of images and videos, their application in precipitation nowcasting and Earth system forecasting is currently in a developmental phase. In many intricate real-world situations, the conditioning information is often highly complex, requiring more than just a few frames. The inherent spatial dynamics and temporal information in prediction further complicate the generation of dynamic video. An interesting question arises: how to capture long-term temporal dependencies within the frame sequence? Recently, large language models (LLMs) have been trained to utilize vast textual datasets, which exhibit remarkable capabilities across diverse tasks surpassing their original linguistic scope. Lian et al. [15] present LLM-ground video diffusion (LVD) that leverages a large language model (LLM) to guide a diffusion model for video generation. Zhang et al. [16] propose Video-LLaMa to understand video content by capturing the temporal changes in visual scenes. In a multi-modal vision–language framework, the integration of the language modality is realized. It is illustrated either by projecting visual tokens to LLMs using linear layers [17,18,19] or by employing cross-attention mechanisms between visual and language tokens [20,21,22]. As we explore the capabilities of LLMs for rainfall forecasts, we utilize a frozen transformer block from pre-trained LLMs as a constituent encoder layer to predict motion trend by understanding long-term temporal context information. It excels at capturing temporal dependencies and the dynamic changes in a high rainfall area between each frame.

In this paper, we propose a two-stage approach named LLMDiff for data-driven precipitation nowcasting. In the first stage of our approach, we train an encoder–decoder conditional network that generates conditional information for the diffusion model. Unlike traditional methods, the network utilizes generated conditional frames to effectively guide the denoising process instead of conditional features. In the second stage of our approach, a denoising network based on Earthformer [7] uses conditional frames, input frames, and noise. This technique aims to reduce the reliance on traditional physical modeling by directly learning from data, offering a data-driven approach that adapts more flexibly to complex weather scenarios. Given the inherent complexities and uncertainties in precipitation nowcasting, this network leverages exceptional capabilities to handle randomness and complexity, capturing the dynamic nature of rainfall events. Furthermore, we incorporate a frozen transformer block from pre-trained LLMs to a denoising network as an encoder layer [23]. By leveraging the capabilities of LLMs, which possess a deep understanding of complex spatiotemporal dynamics between each frame, we guide the diffusion model in estimating the movement tendencies for precipitation nowcasting. Furthermore, the inclusion of LLMs module enhances the generation of sequences with coherent spatial relationships and temporal dependencies. The main contributions of our work can be summarized as follows:

- We propose LLMDiff, a novel model for precipitation nowcasting, which leverages the exceptional capabilities of a diffusion framework based on Earthformer. This structure excels at handling the inherent complexities and uncertainties associated with meteorological conditions, offering a data-driven approach that enhances the prediction of high-quality sequences, closely mirroring real-world atmospheric dynamics.
- We utilize a two-stage method for training an encoder–decoder conditional network and a denoising network. To explore the potential of LLMs in rainfall prediction, the encoder layer of the denoising network includes a frozen transformer block from pre-trained LLMs. Our approach enhances the precision and reliability of precipitation nowcasting predictions.
- LLMDiff significantly outperforms state-of-the-art methods on the precipitation nowcasting benchmark dataset.

## 2. Related Work

### 2.1. Deterministic Predictive Models

**Figure 2:**
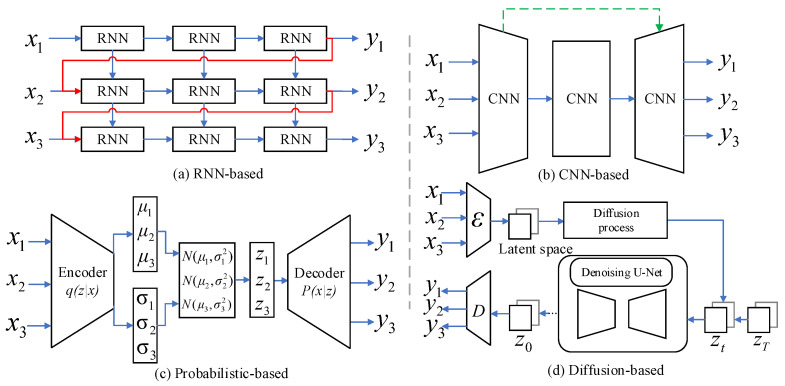
Multiple types of frameworks for precipitation nowcasting. The red lines represent the temporal transition and the green lines capture the spatial dependency. LLMDiff, as part of the diffusion-based architecture, exhibits exceptional performance when compared with other methods.

RNN-based models extract a hidden state from the historical sequence and iteratively produce features utilizing the hidden state. These methods, depicted in Figure 2a, involve the stacking of RNNs for making predictions. For instance, SaGRU [24] introduces a self-attention-based gate recurrent unit model designed to enhance the forecasting of high-impact weather events such as hurricanes and severe convective precipitation. SLTSL [25] captures the distribution and temporal features of precipitation regions from short-term and long-term sequences. Focal frame loss (FFL) [26] introduces a loss function to enhance deep learning models for precipitation nowcasting by focusing on more difficult-to-predict frames within the radar sequence. ConvLSTM [1] expands fully connected LSTMs by incorporating convolutional structures, capturing spatiotemporal correlations. PredRNN [4] proposes a unique convolutional LSTM unit and a spatiotemporal memory cell to construct a predictive recurrent neural network for extrapolations. PhyDNet [5] includes constraints from partial differential equations (PDE) with its recurrent hidden state. E3D-LSTM [27] employs 3D convolution in both the encoding and decoding stages, embedding it into RNNs to capture motion-aware and short-term features.

As illustrated in Figure 2b, CNN-based models process the input frames to create latent representations and generate all predictive frames, rather than using a recurrent method. For example, STACNN [28] integrates multimodal data to enhance the accuracy of precipitation nowcasting, representing a hybrid architecture that combines the spatial processing strengths of CNNs with the temporal dynamics capturing capabilities of RNNs. HTLA [29] combines the strengths of U-Net and transformer through a lightweight attention mechanism, effectively reducing computational complexity and enhancing model efficiency. SimVP [6] is a simple architecture based on existing CNNs, which consists of an encoder, a translator, and a decoder. Earthformer [7], a spatiotemporal transformer for Earth system forecasting, is based on a generic and efficient space–time attention block named cuboid attention. Nevertheless, all deterministic prediction techniques face the challenge of the inherent uncertainty in rainfall systems and high-value echoes fading in precipitation nowcasting due to the disregard of local stochastic factors.

### 2.2. Probabilistic Predictive Models

Predictive models based on probability acquire spatiotemporal uncertainty through the estimation of the conditional distribution of future states. These models are designed to improve the realism of predictions made by variational autoencoders or adversarial training, as shown in Figure 2c.

For example, FsrGAN [30] employs a spatial-channel attention mechanism within an encoder-fusion-decoder architecture, capitalizing on the strengths of both data types to predict small-scale precipitation events more effectively. MultiScaleGAN [31] discusses an improved method for precipitation nowcasting by incorporating adversarial regularization and comparing different types of GANs for the task. SV2P [32], a stochastic variational method for video prediction, generates diverse plausible features for each sample of its latent random variable. Franceschi et al. [33] present a novel stochastic dynamic model designed for video prediction tasks. This model effectively utilizes the structural and computational advantages inherent in state-space models (SSMs) operating on low-dimensional latent spaces. The MoCoGan [34] (motion and content decomposed generative adversarial network) framework generates a video by mapping a sequence of video that consists of a content part and a motion part.

### 2.3. Denoise Predictive Models

In the domain of spatiotemporal prediction, diffusion models have gained significant attention, due to their stable training performance and remarkable capabilities for high-fidelity generation. Recently, many approaches in the field rely on denoising diffusion probabilistic models [14,35] for video prediction, as represented in Figure 2d. For example, Tobias et al. [36] introduce random-mask video diffusion (RaMViD), an extension of image diffusion models to videos using 3D convolutions. During training, a new conditioning technique is introduced, enabling the model to perform video prediction, infilling, and upsampling by varying the mask it conditions on. Yu et al. [37] introduce a new generative model for videos, named the projected latent video diffusion model (PVDM). PVDM is a probabilistic diffusion model designed to learn the video distribution in a low-dimensional latent space, allowing efficient training with high-resolution videos under resource constraints. Yu et al. [38] present Diffcast, an adaptable and comprehensive end-to-end framework that effectively captures the global determinism and local stochastics inherent in precipitation systems. Andrea et al. [39] present the generative diffusion ensemble (GDE) based on the denoising diffusion implicit model (DDIM). The model provides a comprehensive precipitation forecast by training on radar echo datasets.

## 3. LLMDiff

**Figure 3:**
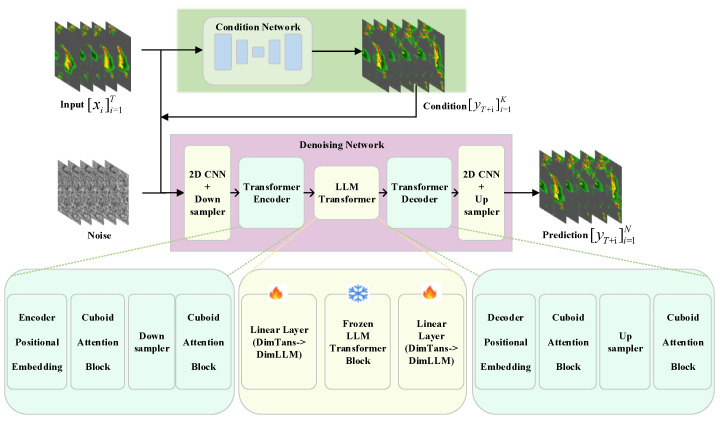
LLMDiff has a novel diffusion structure based on a hierarchical transformer encoder–decoder. *T* is the length of the input sequence, and *K* is the length of the target sequence. The LLM transformer module consists of a frozen transformer block and linear layers, which are used as a visual encoder layer.

This section presents our proposed model, namely LLMDiff. We formulate precipitation nowcasting as a spatiotemporal forecasting problem, similar to the methodologies in [1,13,40,41]. Based on the observation sequence $x_{i}\in R^{H\times W\times C_{in}}$ and diffusion timestep $t\in R^{H\times W\times C_{\mathrm{in}}}$, the model predicts the future $y_{pred}\in R^{H\times W\times C_{out}}$. We denote *H*, *W* as the spatial resolution, and represent $C_{in}$, $C_{out}$ as each space–time coordinate number of the input and the target sequence, respectively. The model can generate varying noise at different diffusion timesteps *t*, contributing to an increase in diversity and quality in predicting the future. We propose a novel diffusion model based on Earthformer shown in Figure 3. Following this introduction, we proceed to offer a detailed analysis of the design aspects for the diffusion model and encoder of a large language model employed in LLMDiff.

### 3.1. Preliminaries

**Expressing diffusion mathematically.** Before presenting our architecture, we provide a summary of the essential concepts that are necessary for comprehending denoising diffusion probabilistic models (DDPMs) [35]. In the context of Gaussian diffusion models, it is assumed that a forward noising mechanism operates, gradually incorporating noise into actual data $x_{0}:q\left(x_{t}|x_{0}\right)=N\left(x_{t};\sqrt{\bar{\alpha }}x_{0},\left(1-\bar{\alpha }\right)I\right)$, in which the constants $\bar{\alpha }$ denote hyperparameters following a fixed schedule. With the application of relevant parameter configurations, $x_{t}=\sqrt{\bar{\alpha }}x_{0}+\sqrt{1-\bar{\alpha_{t}}}\epsilon_{t}$ can be sampled, where $\epsilon_{t}∼N\left(0,I\right)$, $x_{0}∼p\left(x\right)$ are the real data, and $x_{T}∼N\left(0,I\right)$ is random noise.

During the training of diffusion models, performing the reverse process allows for filtering out noise to generate predictions for the future: $p_{\theta }\left(x_{t-1}|x_{t}\right)=N\left(\mu_{\theta }\left(x_{t}\right),\Sigma_{\theta }\left(x_{t}\right)\right)$, where the statistic of $p_{\theta }$ is predicted through the use of neural networks. For spatiotemporal forecasting using diffusion models (DMs), $p_{\theta }\left(x|y\right)$ is expressed as $p_{\theta }\left(x|y\right)=\int p_{\theta }\left(x_{0:T}|y\right)dx_{1:T}=\int p\left(x_{T}\right)\prod_{t=1}^{T}p_{\theta }\left(x_{t-1}|x_{t},y\right)dx_{1:T}$, with $p_{\theta }\left(x_{t-1}|x_{t},y\right)$ serving as the conditional denoising transition conditioned on *y*.

### 3.2. Overall Architecture

To generate predictions that are sharper, clearer, and closer to real-world scenarios, our LLMDiff employs two-stage training approaches: (1) training an encoder–decoder prediction model that generates conditions for the diffusion model; and (2) training a condition DM with frozen LLM transformer block (LLMDiff) which predicts the final future.

#### 3.2.1. Condition Network

It is essential to condition the model in order to guide the diffusion process toward a forecast defined by the known preceding rainfall conditions. In the LLMDiff model, using both input frames and conditional frames simultaneously in the denoising process helps to provide comprehensive spatial–temporal information and guidance to the denoising network, thereby enhancing its predictive power for future precipitation events. Specifically, input frames (typically a series of consecutive radar echo images) contain information about the current and past states of precipitation events, serving as the foundation for forecasting future precipitation trends. However, relying solely on input frames may not adequately capture the complex dynamics and uncertainties of precipitation events. Thus, the introduction of conditional frames is crucial. We acquire conditions in a manner that is both free and simple. Here, we obtain conditions $y_{cond}\in R^{H\times W\times C_{out}}$ by feeding input data $x_{i}\in R^{H\times W\times C_{in}}$ into an encoder–decoder prediction framework (EDNet) [7]. From the input data $x_{i}$, the EDNet predictor produces conditions $y_{cond}$ represented by the following formula:

(1) $$\begin{array}{c} y_{cond}=ED\left(x\right), \end{array}$$

where $H,W,C_{in},C_{out}$ represent the height, width, input channel number, and output channel number, respectively. As shown in Figure 3, we directly provide LLMDiff with the condition information. In particular, the temporal slices of the input data are analogous to color channels in an RGB image. Our model utilizes independent 2D convolutions across these temporal slices, allowing for the extraction of significant frame-level temporal features. This process ensures the generation of a sequence that maintains coherence with the past frames used as conditioning. We utilize instances output by EDNet as conditions, thereby preserving richer spatial information and temporal dependency.

#### 3.2.2. Denoising Design Structure

**Figure 4:**
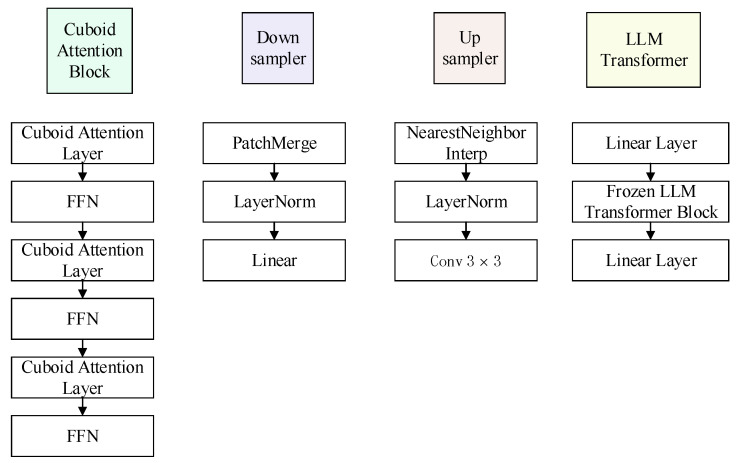
The building blocks of the denoising network used in the LLMDiff model are depicted here. Core components include cuboid attention blocks, 2D CNN layers, upsampling and downsampling units, and a frozen LLM transformer block. These elements work together to process input and handle noise, effectively capturing complex spatiotemporal relationships within the data.

The diffusion model aims to provide a probabilistic approach for generating high-fidelity predictions of future states by gradually adding and then removing noise from the input data. This approach enables the model to capture the inherent uncertainties and complexities associated with the prediction task, resulting in more realistic and accurate forecasts. To ensure superior quality in generating the future, we present a novel diffusion framework based on Earthformer [7]. LLMDiff makes use of the capabilities of diffusion to incorporate the inherent probability distribution of radar echo data. This integration is employed to synthesize a probable precipitation prediction. Assuming that the diffusion model comprehends the stochastic nature of weather dynamics, it can generate a collection of possible forecasting outcomes. The components utilized in the denoising network are shown in Figure 4. The future can be generated by establishing the following distribution:

(2) $$\begin{array}{c} x_{t-1}∼p_{\theta }\left(x_{t-1}|x_{t}\right), \end{array}$$

where $x_{t}$ represents the noisy data point at a certain timestep *t* in the denoising process. It is the current input processed by the model, containing the result of gradually adding noise to the original data. $x_{t-1}$ is the clearer data at the previous timestep that the model attempts to predict. Meanwhile, θ is a set of model parameters learned through training, which define how the model can recover a clearer $x_{t-1}$ from the current noisy data $x_{t}$. In the case of utilizing a T-step denoising diffusion to model the distribution, the formula is shown below:

(3) $$\begin{array}{c} p\left(x_{0:T}|y_{cond}\right)=p\left(x_{T}\right)\prod_{t=1}^{T}p_{\theta }\left(x_{t-1}|x_{t},y_{cond}\right), \end{array}$$

where $y_{\mathrm{cond}}$ represents potential conditional information that can guide the prediction process of the model, making the generated data more consistent with specific contexts. $p(x_{T})$ is the prior distribution of the final noised data $x_{T}$, which is often assumed to be a simple normal distribution, as it represents the state where the data are completely obscured by noise. On the other hand, $p_{\theta }\left(x_{t-1}|x_{t},y_{\mathrm{cond}}\right)$ is the conditional probability distribution of predicting the previous timestep data $x_{t-1}$ given the current noisy data $x_{t}$, conditional information $y_{\mathrm{cond}}$, and model parameters θ. This distribution is continuously optimized through the training process of the model to maximize the joint probability of the entire data sequence $x_{0:T}$ given the conditional information. Thus, noise estimation serves as an optimization objective. We denote the training loss of LLMDiff as the following formula:

(4) $$\begin{array}{c} L=E_{\left(x,y),t,\epsilon ∼N(0,I\right)}||\epsilon -\epsilon_{\theta }\left(x_{t},t,y_{cond}\right){\left|\right|}^{2}, \end{array}$$

where *x* represents the input sequence and *y* denotes the target sequence.

#### 3.2.3. Frozen LLM Transformer Module

Due to the extensive variation and intricacy of Earth’s climatic system, slight variations in the initial setup can result in significantly different outcomes that are challenging to predict. Similarly, the motions observed in rainfall forecasts are extremely complex and exhibit continuous variations in both space and time. Many models focus on capturing basic state transitions across temporal sequences and neglect the motions’ intricate variations, leading to inaccurate predictions in highly dynamic scenarios. According to Wu et al. [42], physical world motions can be naturally decomposed into the transient variation and motion trend. Here, we focus on researching the motion trend, because the natural spatiotemporal processes adhere to the trend dictated by the physics rule. The motion in the video sequence reflects the characteristic attributes of the physical world, such as object inertia, radar echo meteorology, or other physics rules.

To better capture the complex motion trend within space and time, we incorporate a transformer block from pre-trained LLMs as a visual encoder layer to consider temporal dependencies within the frame sequence, as illustrated in Figure 3. Following Pang et al. [23], an untrained LLM transformer block lacks the extensive knowledge and contextual understanding on vast datasets. Furthermore, an untrained LLM transformer block would require significantly more training data and computational resources to achieve a comparable level of performance, increasing the risk of overfitting and reducing the model’s efficiency. Thus, we leverage the prior exposure of the pre-trained transformer block from LLMs like LLaMa-7B [43] to diverse data to rapidly adapt to the specific task of precipitation nowcasting. By freezing the pre-trained weights, the model focuses its learning efforts on refining the diffusion process tailored to radar echo data, ensuring that the LLM transformer block’s inherent strengths in understanding temporal and spatial relationships are fully harnessed. Through a neural network, we encode the input *x* into latent representation *z* and then generate the future frame with an encoder $F_{E}$ and decoder $F_{D}$,

(5) $$\begin{array}{c} F_{E}\left(x\right)\to z,F_{D}\left(z\right)\to y. \end{array}$$

Between the encoder $F_{E}$ and decoder $F_{D}$, we introduce a single pre-trained transformer block denoted as $F_{LM}$, sourced from an LLM such as LLaMa. Addressing the diverse feature dimensions between the encoder $F_{E}$ and the language transformer $F_{LM}$, two linear layers ($F_{L}^{1}$ and $F_{L}^{2}$) are applied before and after $F_{LM}$ to align the dimensionality. The equation is expressed as follows:

(6) $$\begin{array}{c} F_{E}\left(x\right)\to z, \end{array}$$

(7) $$\begin{array}{c} F_{L}^{1}\left(z\right)\cdot F_{LM}\cdot F_{L}^{2}\to z^{\prime }, \end{array}$$

(8) $$\begin{array}{c} F_{D}\left(z^{\prime }\right)\to y. \end{array}$$

The training stage involves keeping the pre-trained transformer $F_{LM}$ frozen, but all other modules are trained as usual including $F_{L}^{1}$ and $F_{L}^{2}$.

In the construction of the LLMDiff model, supervised fine-tuning represents a pivotal step. By training on a labeled dataset, the model optimizes its parameters to more accurately predict precipitation patterns. Specifically, we first train a conditional encoder-decoder network that ingests a series of historical radar echo images as input and generates conditional information frames, which subsequently guide the prediction process of the denoising network. Subsequently, the denoising network utilizes these conditional frames, input frames, and added noise to iteratively denoise and reconstruct future radar echo images. During this process, we employ a frozen transformer block from a pre-trained large language model (LLM) as a general-purpose visual encoder layer within the denoising network, enabling it to capture long-range temporal context information and accurately estimate the trends of precipitation movement. The entire model undergoes supervised fine-tuning, minimizing the discrepancies between predicted and real images to continuously enhance prediction accuracy, ultimately demonstrating exceptional performance in the task of precipitation nowcasting.

### 3.3. Parameter Settings for Model Architecture

The parameter settings for the LLMDiff model structure are as follows: In the encoder section, several Conv2d layers are used, with the input and output channels set to (16, 32), (32, 64), and (64, 128) respectively, a kernel size of 3 × 3, and a stride of 1. Each convolutional layer is followed by GroupNorm for normalization and LeakyReLU for nonlinear activation. The PatchMerging 3D layer is used to reduce spatial dimensions, increasing the input channels from 128 to 256, followed by LayerNorm for further normalization. The core part of the model is the cuboid transformer encoder, which integrates multiple stack cuboid attention modules. Each block has input and output dimensions of (256 and 512) and uses linear layers (from 512 to 1024), the GELU activation function, and dropout (with a probability of 0.1) for regularization. The cuboid transformer decoder has a similar structure to the encoder but includes a cross-attention mechanism, progressively reducing dimensions from 512 to 256 to enhance the reconstruction capability of the output.

The LLM transformer module is used for processing sequence data, with an embedding size of 1024. The linear layers are set to dimensions (1024 and 2048), and RMSNorm is used for normalization. The dropout rate is set to 0.1 to ensure the model’s generalization ability. The entire model’s parameters have been carefully tuned. In the LLM transformer module, the model employs a multi-layer stacked self-attention mechanism, with each attention head dimension set to (1024, 2048), and uses linear layers to reduce the output dimension to 512, which is then output to the decoder. During decoding, a structure similar to the encoder is used, with a PatchExpanding layer to gradually restore spatial dimensions, ultimately reducing the number of channels to the size of the original image. Additionally, the LLMDiff model incorporates a noise prediction conditional diffusion model, which progressively denoises to generate accurate predicted images. All convolutional and linear layers are initialized to ensure stable convergence during training.

## 4. Experiments

In this section, we first evaluate the effectiveness of LLMDiff and carry out an ablation study on our proposed model using the SEVIR dataset (size of 384 × 384) [41]. Our proposed method LLMDiff achieves better performance, while handling the issue of the inherent uncertainty in rainfall systems and inaccurate estimation of motion trend. Table 1 displays the statistics for the dataset employed in the experiments. The normalization process involves mapping the value of pixel to the range [0, 1].

### 4.1. Dataset

**Figure 5:**
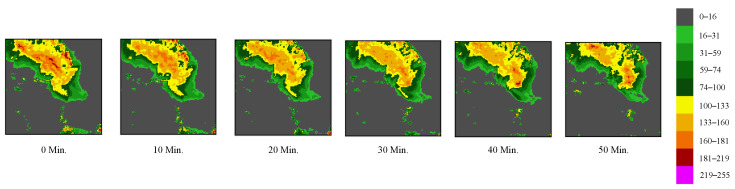
The storm event imagery (SEVIR) dataset provides some examples of a vertically integrated liquid (VIL) observation sequence. A higher precipitation intensity is indicated by a larger pixel value within the mapped range of 0–255 for observation intensity.

A spatiotemporal Earth observation dataset, SEVIR (storm event imagery) [41], consists of image sequences extending over 384 km × 384 km and spanning a duration of 4 h. Within SEVIR, images are sampled and synchronized across five distinct data types: three channels (C02, C09, C13) from the GOES-16 advanced baseline imager, NEXRAD vertically integrated liquid (VIL) mosaics (as shown in Figure 5), and flashes identified by the GOES-16 geostationary lightning mapper (GLM) flashes. Researchers can utilize the SEVIR benchmark to explore multiple meteorological applications, such as precipitation nowcasting, synthetic radar generation, and front detection. Our goalis to predict the future VIL up to 60 min (6 frames), utilizing a context of 70 min of VIL (7 frames). We perform all experiments on machines equipped with NVIDIA A100 GPUs.

### 4.2. Evaluation Metric

The precision of nowcasting is evaluated by computing the mean critical success index (CSI) [7], mean squared error (MSE) and the continuous ranked probability score (CRPS) [44]. Similar to IoU (intersection over union), the CSI value is employed to measure the extent of pixel-wise alignment between predictions and ground truth, obtained by thresholding them into 0/1 matrices and the formula is as follows:

(9) $$\begin{array}{c} CSI=\frac{\neq Hits}{\neq Hits+\neq Misses+\neq F.Alarms}. \end{array}$$

To compute the ≠Hits (truth = 1, pred = 1), ≠Misses (truth = 1, pred = 0), and ≠FalseAlarms (truth = 0, pred = 1), the prediction and ground truth undergo rescaling to the 0–255 range and binarization using threshold [16, 74, 133, 160, 181, 219]. The CSI values are calculated at different thresholds, and the mean CSI-M is included for summarization.

The continuous ranked probability score (CRPS) value is a commonly employed metric in weather and climate forecasting for measuring forecast accuracy [45]. CRPS evaluates forecasts based on the full set of ensemble predictions. For each pixel in a forecasted image, CRPS is calculated as the integral of the squared difference between the cumulative distribution function (CDF) of the forecast ensemble and the CDF of the observations. The observation CDF is represented as a Heaviside step function *H* at the point $x_{\mathrm{true},i}$. The CRPS value for the pixel *i* is calculated as follows:

(10) $$\begin{array}{c} \mathrm{CRPS}=\int_{-\infty }^{\infty }{\left(F\left(x^{\prime }\right)-H\left(x^{\prime }-x_{\mathrm{true},i}\right)\right)}^{2}dx^{\prime }. \end{array}$$

For an entire image, the CRPS value is the average across pixel-level CRPS scores. Thus, CRPS can be regarded as an extension of the MAE for probabilistic forecasting, enabling simultaneous comparison between ensemble and deterministic predictions. We utilize both average-pooling and max-pooling to analyze average and maximum values within local neighborhoods by employing neighborhood sizes of 4 × 4 (stride 2) and 16 × 16 (stride 4) [46].

### 4.3. Comparison Analysis

We trained for 100 epochs on the dataset and used early-stop model training according to the validation score with a tolerance of 20. We adopted a 20 % linear warm-up and Cosine learning rate scheduler that decays the learning rate from its maximum to zero after warm-up. We adopted parallel dataand gradient accumulation to use a total batch size of 64.

**Figure 6:**
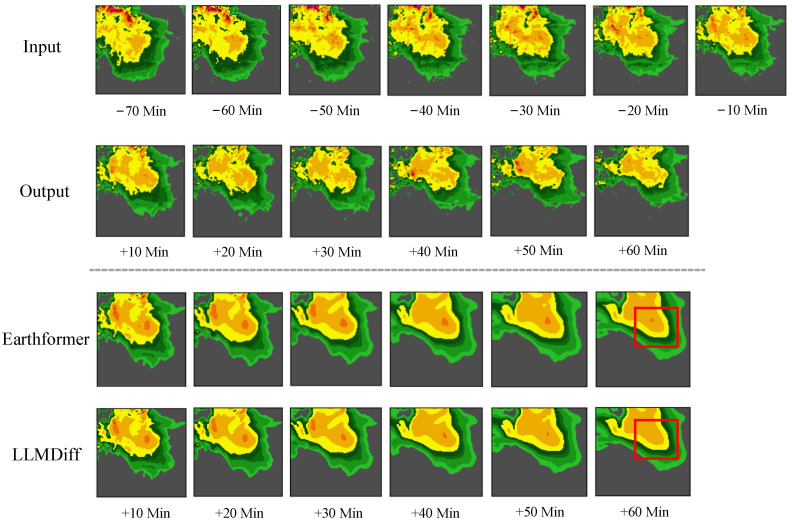
A set of example forecasts from SEVIR is presented, including those from Earthformer and LLMDiff. From top to bottom: input sequence, target sequence, predictions from Earthformer and LLMDiff.

We chose the following spatiotemporal forecasting algorithms for comparison: Unet [47], ConvLSTM [1], PredRNN [4], PhyDNet [5], E3D-LSTM [27], Rainformer [48], and Earthformer [7]. In Table 2, we conduct experiments to compare our proposed model LLMDiff with state-of-the-art models. Table 2 shows a significant improvement of approximately 5.32% in CSI-160, with the index value moving from 0.3138 to 0.3305. The most considerable increase noted for CSI-219 is 8.18%, with the value enhancing from 0.1675 to 0.1812. Across almost all metrics, LLMDiff consistently performs better than the other models, delivering notable performance enhancements, especially at higher thresholds such as CSI-160 and CSI-219. Figure 6 shows that LLMDiff outperforms the baseline with predictions of enhanced quality and more precise localization. Especially when generating the prediction for the 60th minute, the LLMDiff model’s superior capabilities are more clearly showcased. As shown in the red box in Figure 6, the predictions of LLMDiff are closer to the truth.

The backbone network in our framework is Earthformer, which we use as the baseline for our comparison. Additionally, we choose to compare with other state-of-the-art models. For example, ConvLSTM and PredRNN based on the RNN structure generate frames sequentially. The results of our experiments are presented in Table 2. A conclusion can be inferred from the content of Table 2. First, our proposed LLMDiff has shown a significant enhancement compared with the backbone, with enhancements ranging from 2% to 10% in terms of the CSI threshold. The results confirm that our framework is effective in improving the prediction accuracy of backbone. Secondly, when the CSI threshold is set to 16, our proposed LLMDiff falls short of optimal performance. It is evident that modeling the precipitation system with global motion trends and local stochastics is more effective in modeling the precipitation system than treating the entire system as stochastic.

The LLMDiff model performs the best in terms of CRPS, with a value of 0.0245, indicating high accuracy in its probability predictions. Following closely, the Rainformer and Earthformer models also have relatively low CRPS values, both at 0.0251, demonstrating good predictive accuracy. Not only do the LLMDiff, Rainformer, and Earthformer models excel in CRPS, but they also generally perform well across various CSI indicators, showcasing their strong capabilities in handling forecasting tasks.

### 4.4. Ablation Study

According to Table 3, the ablation study was designed to discern the utility and impact of each component on our model’s overall performance, allowing us to identify indispensable elements and potential areas for optimization. This component of diffusion was crucial for initial data preprocessing and feature extraction. Its removal led to a significant degradation in model performance, with accuracy dropping by approximately 15%. This underlines its pivotal role in setting the stage for effective data analysis. The exclusion of a frozen transformer block from LLMs had a moderate impact, with a 5% decrease in overall performance. This suggests that while the component contributes to enhancing model functionality, it does not perform core processing tasks.

**Figure 7:**
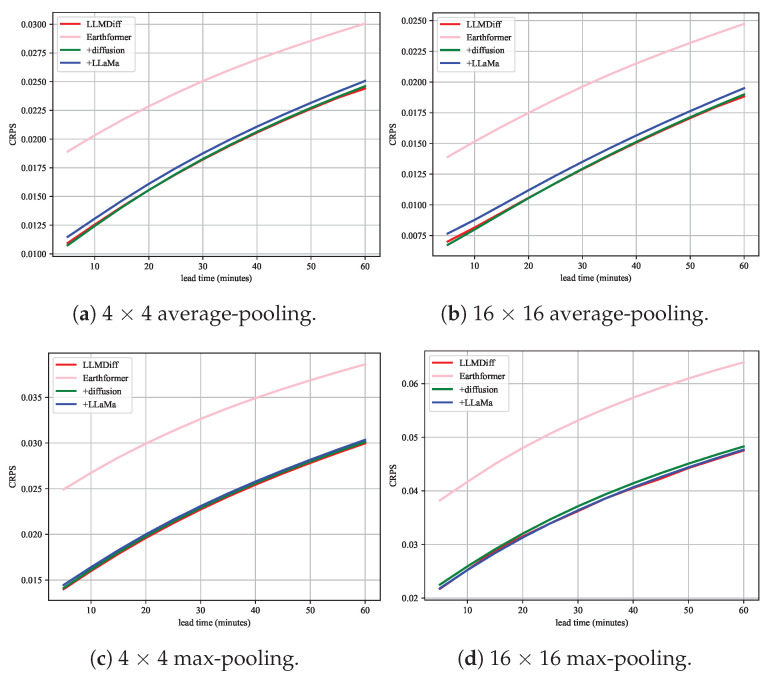
Changes in CRPS over different nowcasting time intervals across various spatial neighborhood configurations.

The results displayed in Figure 7 from the ablation studies provide critical insights into the individual contributions of various components within the LLMDiff model. Figure 7 compares the CRPS across four different model configurations with varying lead time and pooling strategies, emphasizing the effect of each architectural element on the model’s probabilistic forecasting performance. LLMDiff consistently shows lower CRPS values across all configurations and lead time, suggesting its superior performance in probabilistic forecasting compared to the other models. LLMDiff with all components integrated performs the best in terms of CRPS, indicating the highest accuracy in probabilistic predictions. The CRPS values for models incorporating the diffusion component are consistently lower. The results suggest that the diffusion component not only contributes to noise reduction and data reconstruction during the denoising process but also enhances the overall predictive capability by effectively capturing the inherent uncertainties of the weather dynamics. The inclusion of the LLaMa component, as part of the LLMDiff model, shows a marked improvement in CRPS across various lead time and pooling configurations. This highlights its effectiveness in refining the model’s probabilistic forecasting capabilities. Earthformer performs worse, underscoring the importance of these features in achieving lower prediction errors.

## 5. Conclusions

In this paper, we propose LLMDiff, a novel diffusion model for precipitation nowcasting. LLMDiff consists of a two-stage training approach for deep learning in Earth system forecasting. Specially, in order to explore the potential of LLMs for rainfall prediction, we introduce a transformer block from LLMs as a visual encoder layer that is capable of considering long-term temporal context information and capturing temporal dependencies within the frame sequence. Our method has demonstrated significant performance on the SEVIR dataset, as shown by our experiments. Significant future efforts are required to enhance the precision of precipitation nowcasting. One potential approach involves incorporating additional physical principles, such as motion trend. Alternatively, exploring a wider range of meteorological data, including satellite observations, could also lead to improvements. We anticipate that this research will motivate future research in these directions.

## Figures and Tables

**Table 1:** Dataset used and its statistical configuration.

Dataset	Size			Seq. Len		Spatial Resolution
	Train	val	Test	in	out	H×W
SEVIR	35,718	9060	12,159	13	12	384 × 384

**Table 2:** Performance comparison with the proposed method LLMDiff on SEVIR. We used critical success index (CSI), continuous ranked probability score (CRPS) and mean squared error (MSE) as the evaluation metrics. According to intersection over union (IOU), the CSI value is determined at different precipitation thresholds and labeled as CSI-thresh. CRPS is an indicator for evaluating the quality of probability predictions, where a lower value indicates more accurate prediction. “\" indicates missing data. The best-performing variant is denoted in bold.

Model	CSI-M↑	CSI-219↑	CSI-181↑	CSI-160↑	CSI-133↑	CSI-74↑	CSI-16↑	MSE↓	CRPS↓
Unet	0.3593	0.0577	0.1580	0.2157	0.3274	0.6531	0.7441	4.1119	\
ConvLSTM	0.4185	0.1288	0.2482	0.2928	0.4052	0.6793	0.7569	3.7532	0.0264
PredRNN	0.4080	0.1312	0.2324	0.2767	0.3858	0.6713	0.7507	3.9014	0.0271
PhyDNet	0.3940	0.1288	0.2309	0.2708	0.3720	0.6556	0.7059	4.8165	0.0253
E3D-LSTM	0.4038	0.1239	0.2270	0.2675	0.3825	0.6645	0.7573	4.1702	\
Rainformer	0.3661	0.0831	0.1670	0.2167	0.3438	0.6585	0.7277	4.0272	\
Earthformer	0.4343	0.1675	0.2815	0.3138	0.4201	0.6845	0.7385	3.6692	0.0251
LLMDiff	**0.4508**	**0.1812**	**0.2817**	**0.3305**	**0.4313**	**0.6956**	**0.7576**	**3.5581**	**0.0245**

**Table 3:** Ablation studies with the proposed diffusion structure and large language models (LLMs) on SEVIR. The best-performing variant is denoted in bold.

Model	Metrics							
	CSI-M↑	CSI-219↑	CSI-181↑	CSI-160↑	CSI-133↑	CSI-74↑	CSI-16↑	MSE↓
Earthformer	0.4343	0.1675	0.2815	0.3138	0.4201	0.6845	0.7385	3.669
+diffuion	0.4336	0.1757	0.2782	0.3171	0.4242	0.6863	0.7512	3.6352
+LLaMa	0.4239	0.1369	0.2549	0.2989	0.4066	0.6820	0.7539	3.6633
LLMDiff	**0.4508**	**0.1812**	**0.2817**	**0.3305**	**0.4313**	**0.6956**	**0.7576**	**3.5581**

## Data Availability

Code and data to reproduce our experiments are available at https://github.com/LeiShe1/LLMDiff.
